# Diagnosis of Extensive DCIS with Progressive Microcalcifications over a Period of 11 Years

**DOI:** 10.3390/diagnostics16172819

**Published:** 2026-09-02

**Authors:** Menelaos Zafrakas, Theodoros Argyriou, Panayiota Papasozomenou, Christos Emmanouilides

**Affiliations:** 1 School of Health Science, International Hellenic University, 57400 Thessaloniki, Greece; 2 Department of Medical Oncology, European Interbalkan Medical Center, 55535 Thessaloniki, Greece

**Keywords:** ductal carcinoma in situ, DCIS, microcalcifications

## Abstract

A 53-year-old woman presented complaining of enlargement of her right breast; 12 years earlier she was treated elsewhere for an invasive ductal carcinoma of the right breast with breast-conserving surgery, adjuvant chemotherapy, anti-HER2 therapy, radiotherapy and endocrine therapy. One year postoperatively, a group of microcalcifications was seen in the ipsilateral breast on mammography. In the following years, the area of microcalcifications progressively increased, but instead of vacuum-assisted biopsy, the treating physicians performed core needle biopsies that were negative for malignancy. Twelve years after initial diagnosis, when we first examined the patient, the right breast was diffusely hard on palpation and larger than the left. On ultrasound, a large area of architectural distortion was seen in the lower-inner quadrant; after core needle biopsy, histology showed extensive fibrosis with almost complete absence of cells. Due to the progressive enlargement of the area of microcalcifications, simple mastectomy with sentinel lymph node biopsy was performed. Final histology showed extensive areas of high-grade ductal carcinoma in situ (DCIS), pTis, pN0. This case highlights the long period of time that DCIS may exist without progression to invasive disease and possible caveats that may delay diagnosis of DCIS with potentially serious medicolegal implications.

**Figure 1:**
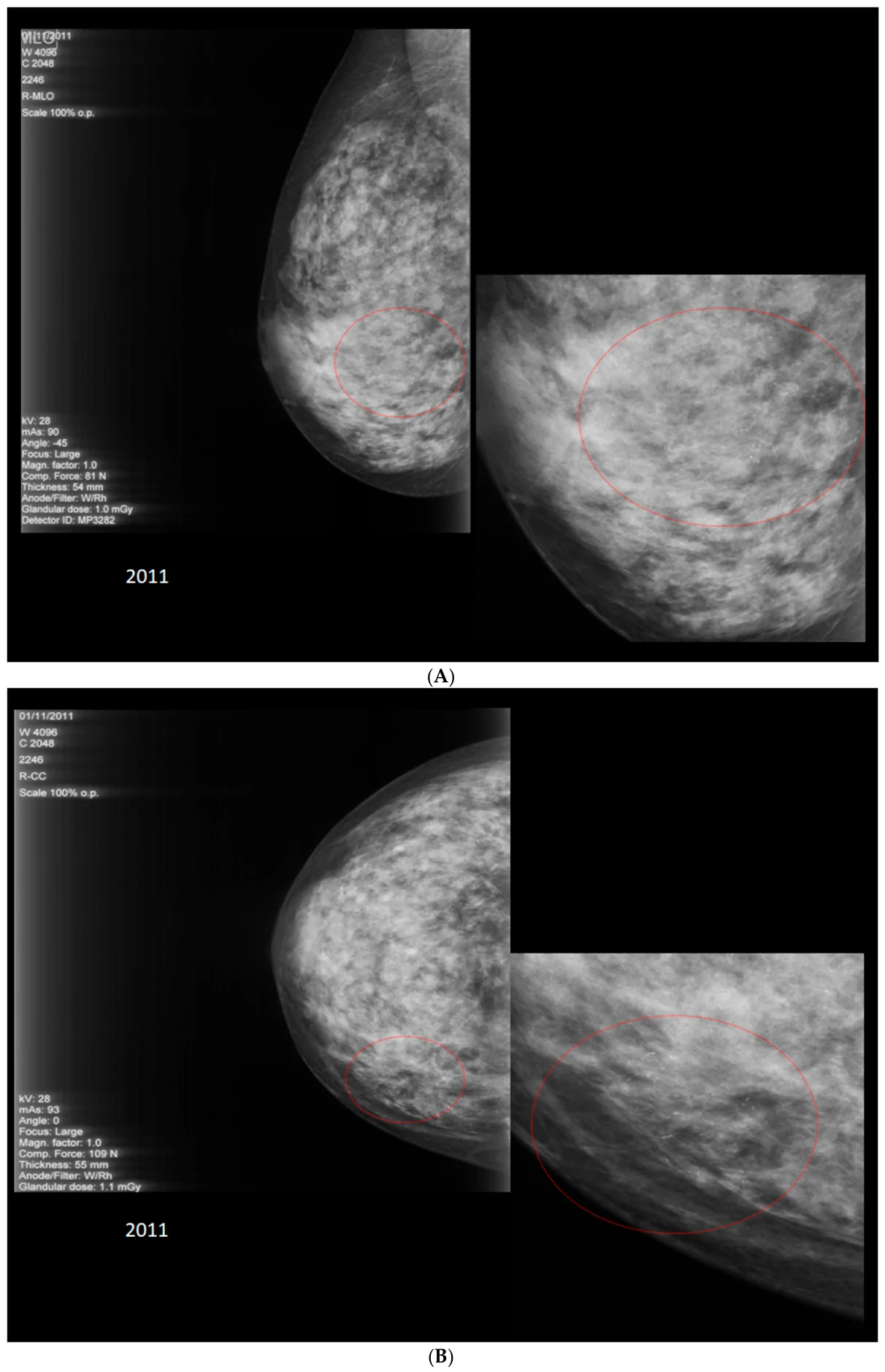
(**A**) Breast microcalcifications are deposits of calcium in the breast tissue, appearing as small bright spots on mammograms [1,2]. Microcalcifications are present in approximately one-third of all malignant lesions detected at screening mammography [2,3,4]. Microcalcification clusters are an independent risk factor for breast cancer, with a higher estimated risk in premenopausal women, and they are significantly associated with ductal carcinoma in situ (DCIS) [2]. DCIS diagnosis is often challenging, especially for low-grade lesions [5] and lesions lacking microcalcifications [6]. Vacuum-assisted biopsy (VAB) is widely recognized as the most appropriate minimally invasive diagnostic procedure for breast microcalcifications, since much larger tissue samples can be obtained compared with core needle biopsy (CNB) [7]. Yet, if microcalcifications are accompanied by other findings suggestive of invasive cancer, e.g., a hypoechoic mass or architectural distortion, CNB may be the preferred method, as it is less invasive, more widely available, and less expensive. Herein, we present a case of DCIS with an exceptionally long and well-documented radiological follow-up demonstrating the progressive evolution of mammographic microcalcifications over more than a decade before the diagnosis of extensive high-grade DCIS; in this case, CNB was used instead of VAB, and diagnosis was probably delayed, with potentially serious medicolegal consequences. A 53-year-old woman presented complaining of enlargement of her right breast accompanied by mild mastalgia. We examined the patient for the first time 12 years after the diagnosis of invasive ductal carcinoma of the right breast, at the age of 41; pT1c, pN0, M0, G2, R0, ER-positive, PR-positive, HER2-positive (3+), Ki67 at 40%. The patient was treated back then elsewhere with wide local excision and sentinel lymph node biopsy, followed by adjuvant chemotherapy and targeted anti-HER2 therapy, radiotherapy, and endocrine therapy with tamoxifen for seven years. One year postoperatively, a group of microcalcifications was noted in the ipsilateral breast. This figure shows medio-lateral oblique (MLO) mammographic images of the right breast, taken one year postoperatively, when the patient was 42 years old, showing a group of microcalcifications (in the red circles); the right-hand image is a magnification of the image on the left. (**B**) Cephalo-caudal (CC) mammographic images of the right breast taken one year postoperatively, when the patient was 42 years old, showing a group of microcalcifications (in the red circles); the right-hand image is a magnification of the image on the left.

**Figure 2:**
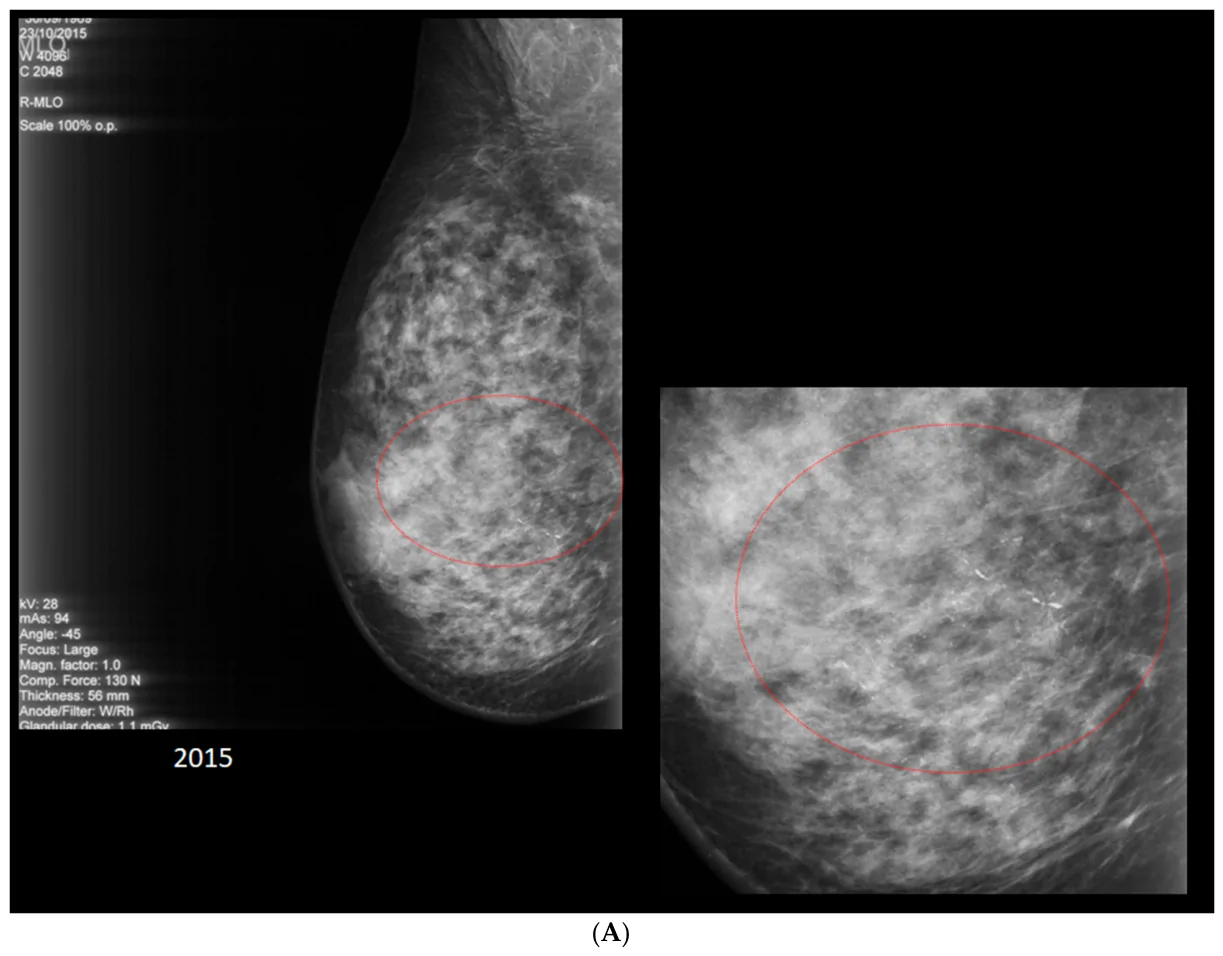

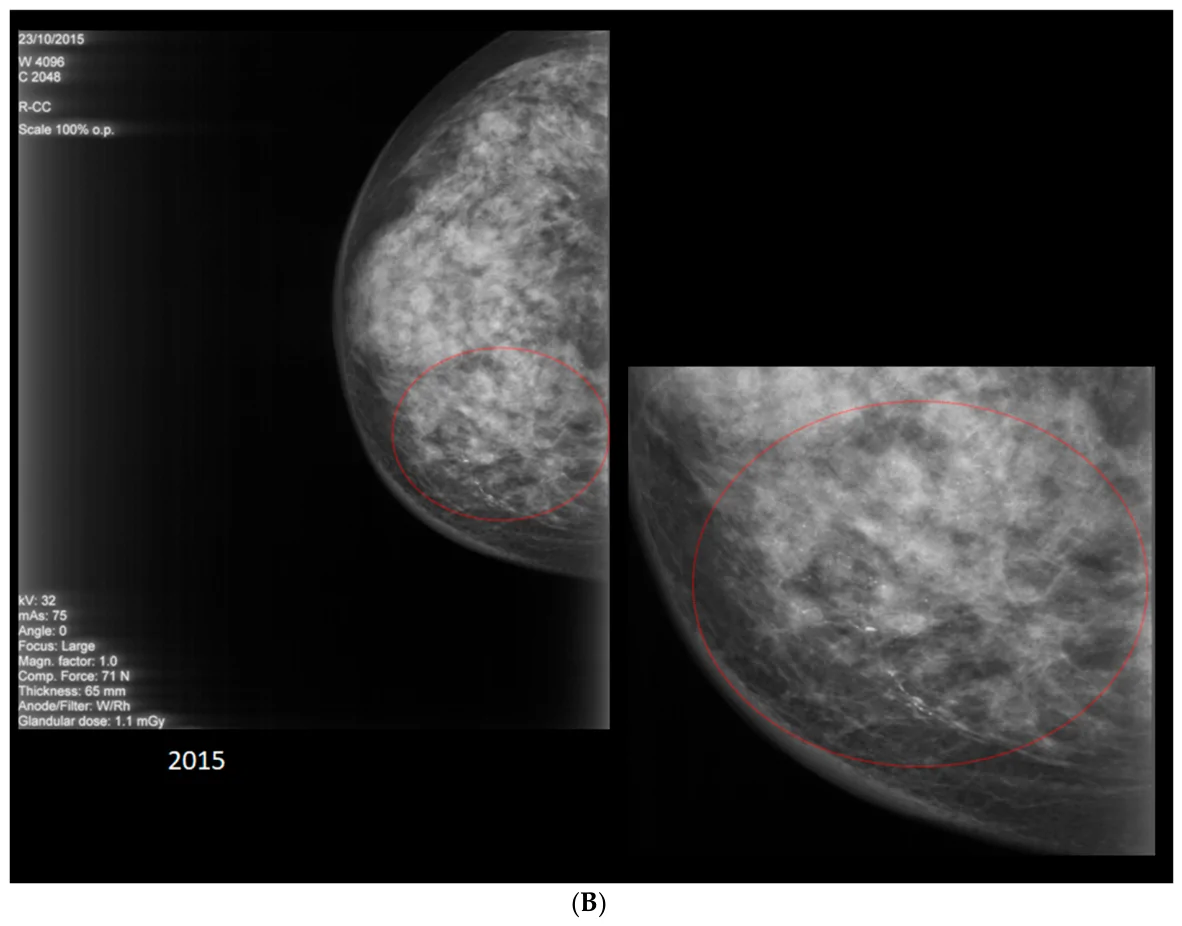
(**A**) Medio-lateral oblique (MLO) mammographic images of the right breast, taken five years postoperatively, when the patient was 46 years old, showing a now larger group of microcalcifications (in the red circles); the right-hand image is a magnification of the image on the left. (**B**) Cephalo-caudal (CC) mammographic images of the right breast taken five years postoperatively, when the patient was 46 years old, showing a now larger group of microcalcifications (in the red circles); the right-hand image is a magnification of the image on the left.

**Figure 3:**
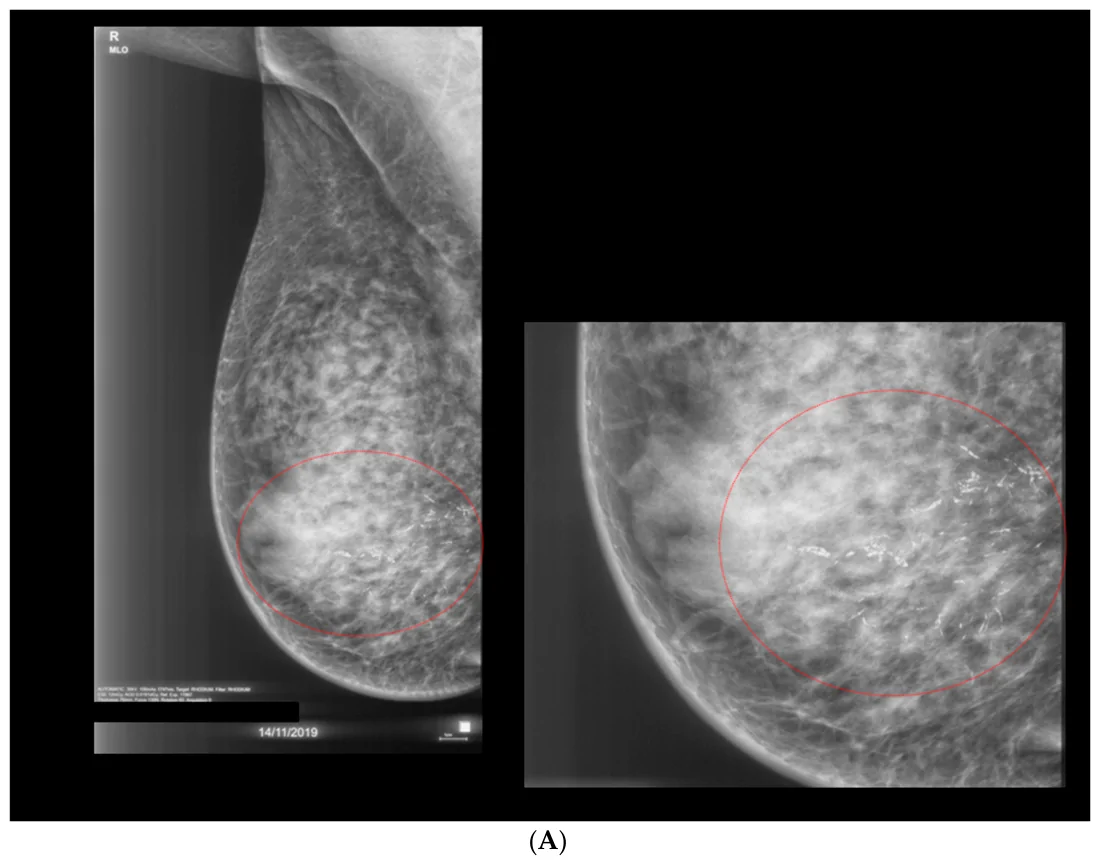

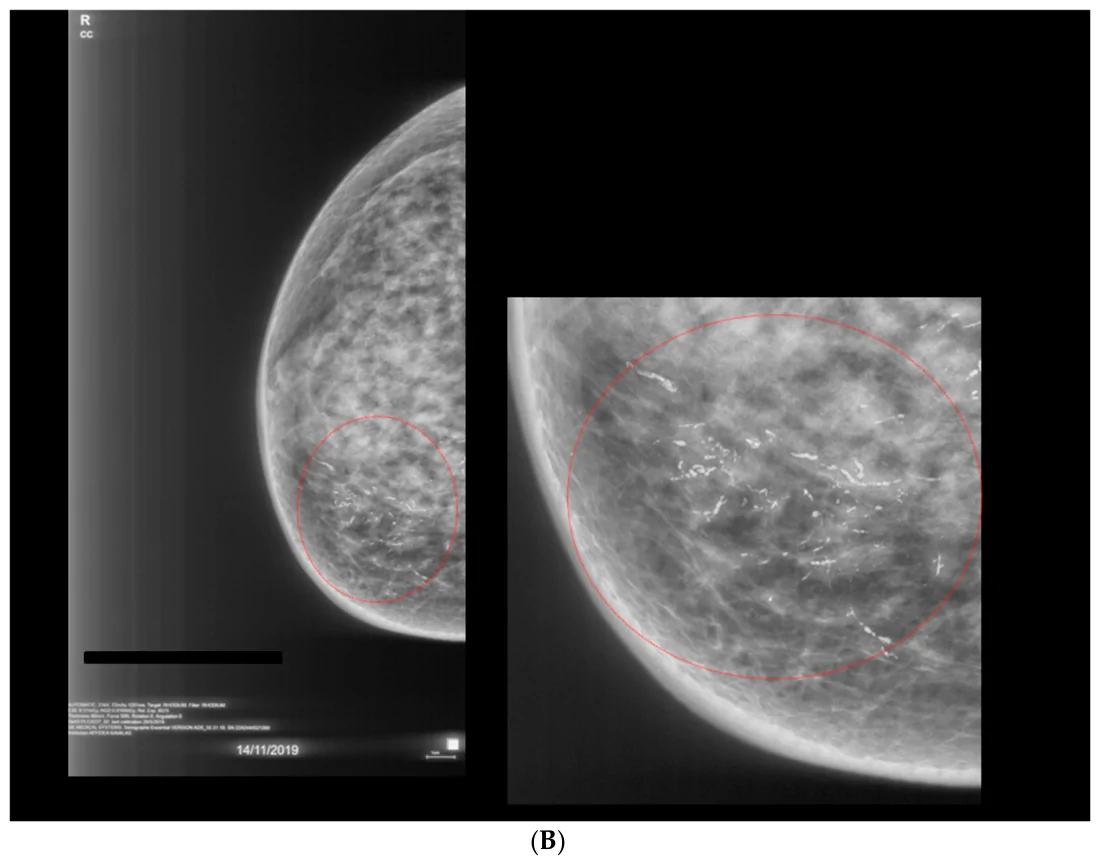
(**A**) Medio-lateral oblique (MLO) mammographic images of the right breast, taken eight years postoperatively, when the patient was 49 years old, showing an even larger group of microcalcifications (in the red circles) compared with previous mammograms; the right-hand image is a magnification of the image on the left. (**B**) Cephalo-caudal (CC) mammographic images of the right breast taken eight years postoperatively, when the patient was 49 years old, showing an even larger group of microcalcifications (in the red circles) compared with previous mammograms; the right-hand image is a magnification of the image on the left.

**Figure 4:**
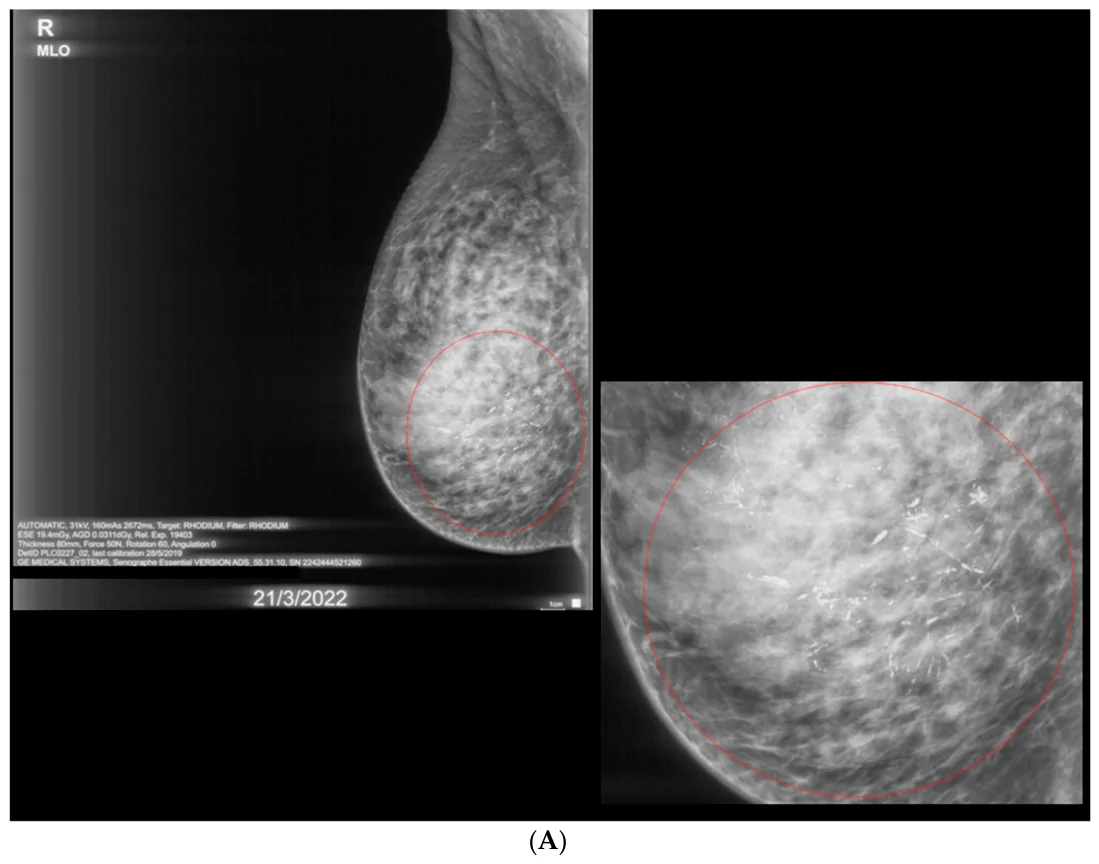

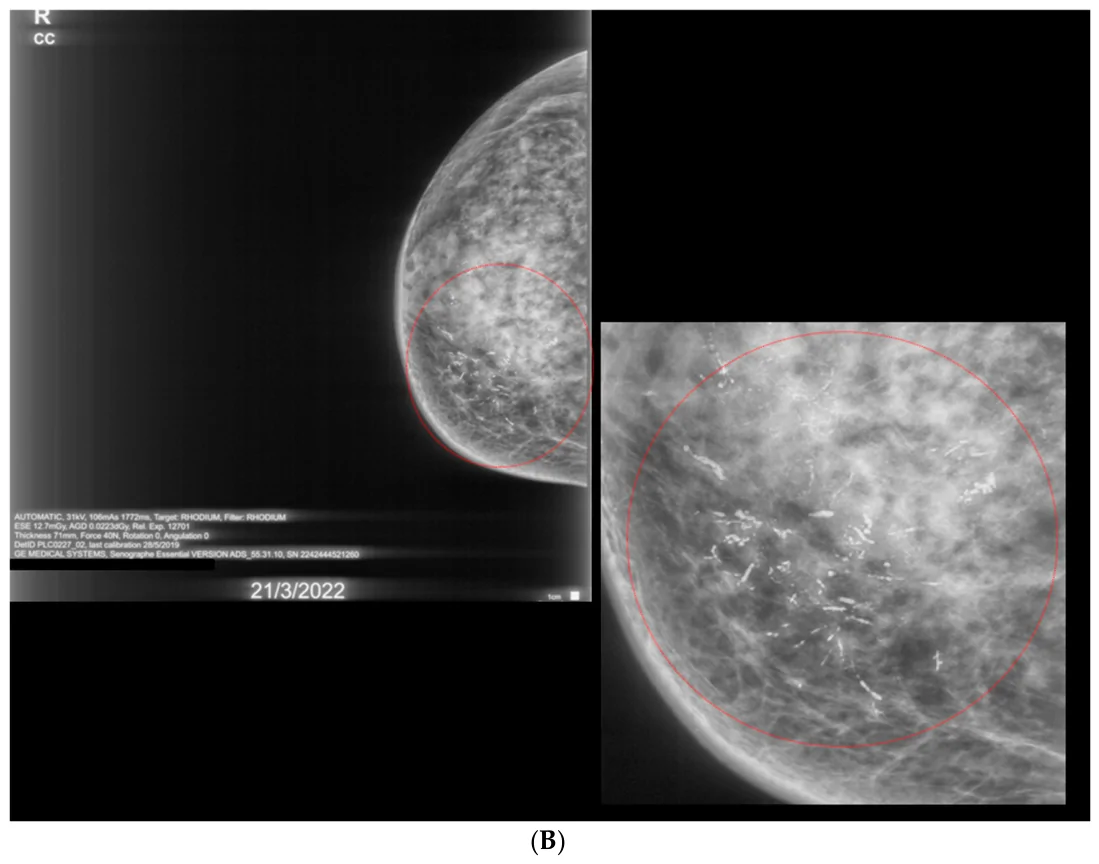
(**A**) Medio-lateral oblique (MLO) mammographic images of the right breast, taken 12 years after initial diagnosis, when the patient was 53 years old, showing now extended microcalcifications (in the red circles); the right-hand image is a magnification of the image on the left. (**B**) Cephalo-caudal (CC) mammographic images of the right breast taken 12 years after initial diagnosis, when the patient was 53 years old, showing now extended microcalcifications (in the red circles); the right-hand image is a magnification of the image on the left.

**Figure 5:**
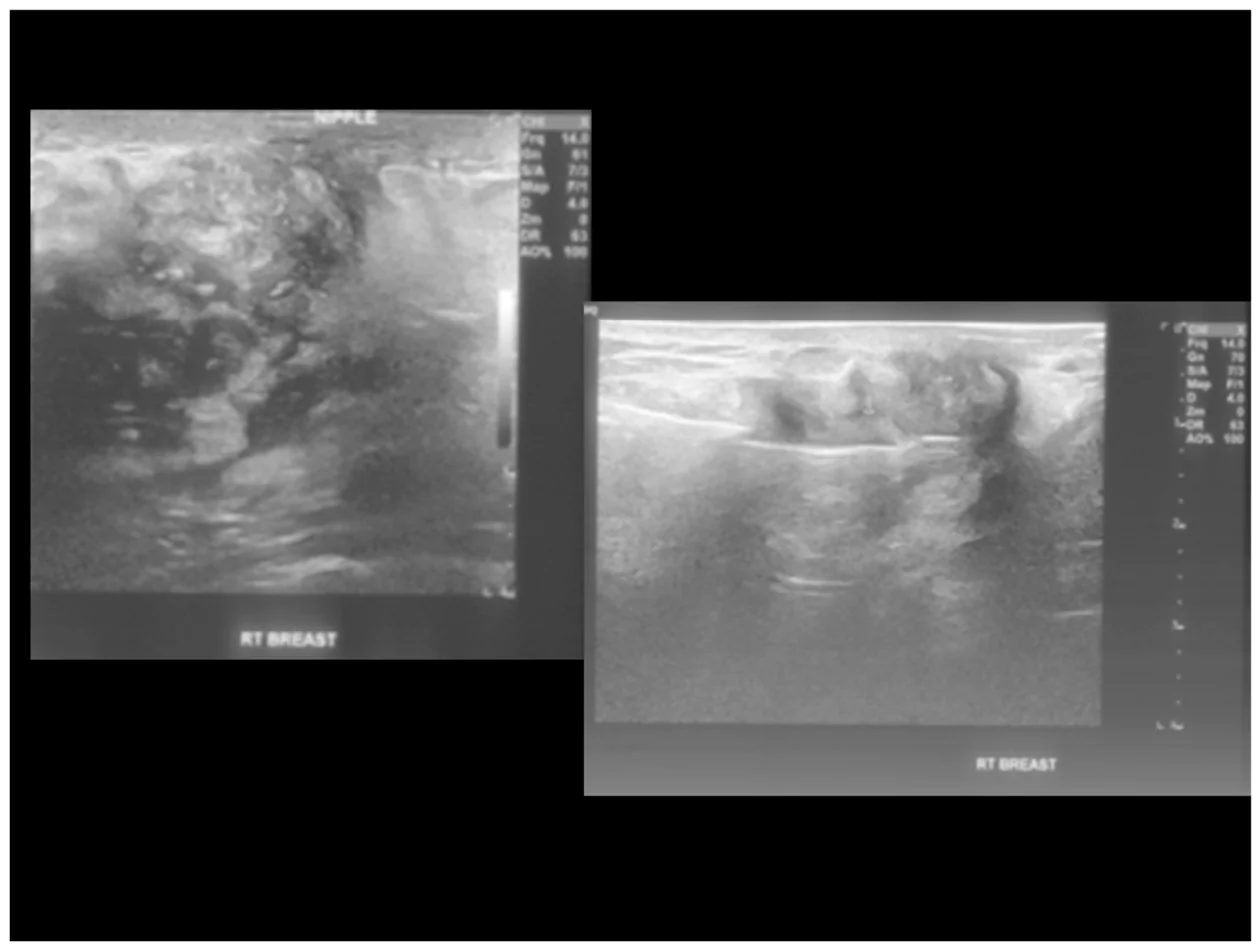
We examined the patient for the first time 12 years after the diagnosis of invasive ductal carcinoma of the right breast. After extensive discussion with the patient, we concluded that she was well aware that the area of microcalcifications was progressively increasing. She had undergone repeated MRIs which did not show any signs suspicious of disease recurrence, and three core needle biopsies (CNBs) under ultrasound guidance which were all negative for malignancy. Upon clinical examination, we noted mild breast asymmetry, with the right breast larger than the left; a surgical scar was noted in the upper inner quadrant of the right breast; the right breast was diffusely hard on palpation. On ultrasound, a large area of architectural distortion was seen (here shown in the left-hand image) in the inner-lower quadrant of the right breast. These sonographic findings were very prominent, so that after extensive discussion with the patient, CNB was performed under ultrasound guidance (shown in the right-hand image), despite previous negative CNBs; histology showed extensive fibrosis with almost complete absence of cells. Despite negative histology, based on the progressive enlargement of the area of microcalcifications, the low sensitivity of CNB, the deteriorating clinical presentation, and upon the patient’s request, simple mastectomy was performed together with repeat sentinel lymph node biopsy (SLNB), because if invasive disease had been diagnosed after mastectomy, then a SLNB would no longer be feasible. Final histology showed extensive areas of high-grade ductal carcinoma in situ (DCIS), mainly of comedo type with small proportions of solid type and cancerization of lobules; estrogen receptor-negative, progesterone receptor-negative, with negative SLNB, pTis, pN0, R0. It is worth noting that DCIS foci were found throughout the mastectomy specimen, and not only in the area of the mammographic microcalcifications and the sonographic architectural distortion (i.e., in the lower-inner quadrant) nor in the area of the first operation (i.e., the upper inner quadrant). All interventions were carried out after detailed counseling and informed consent of the patient. Postoperative testing with next-generation sequencing was negative for BRCA1 and BRCA2 germline pathogenic mutations. The patient remains disease-free four years post-mastectomy and 16 years after initial diagnosis of invasive breast cancer; yet she did not raise any claims of medical liability for delayed diagnosis.

**Figure 6:**
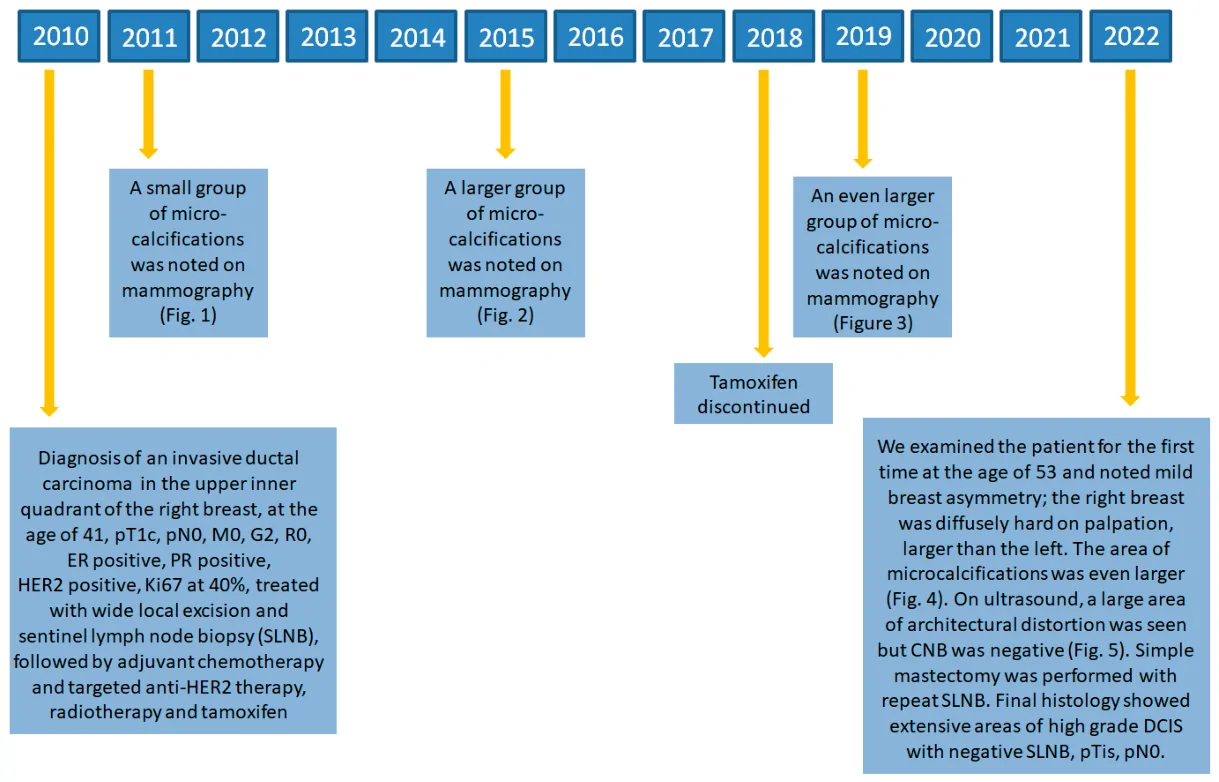
A timeline showing an overview of the clinical course of the patient diagrammatically. This case highlights potential caveats that may delay diagnosis of DCIS with potentially serious medicolegal implications; in particular, it is well established that for DCIS breast ultrasound and breast MRI have lower sensitivity than mammography and core needle biopsy has lower sensitivity than VAB [7,8,9,10,11,12]. In the case of breast microcalcifications, stereotactic rather than ultrasound-guided biopsy and vacuum-assisted biopsy (VAB) rather than CNB are the most reliable diagnostic methods [13,14]; the calcified areas should be targeted adequately, removal of microcalcifications should be confirmed with mammography of the breast specimen, and radiologic-pathologic concordance should be assessed after each biopsy procedure [15,16]. Otherwise, diagnosis may be delayed significantly, and DCIS may progress even to invasive disease, potentially leading to serious medical liability claims against the treating physicians. Furthermore, this case demonstrates the importance of clinical judgment in cases of persistent radiological suspicion, despite repeatedly negative pathology results. A careful overall assessment of all available clinical, imaging, and pathological findings is of paramount importance for clinical decision-making regarding the therapeutic management of such cases. This case also highlights the long period of time that DCIS may exist without progression to invasive disease and the complexity of the natural history of DCIS [17,18,19,20]. Interpretation of these findings should be made cautiously, as this is a single case of a patient previously treated with adjuvant chemotherapy, targeted anti-HER2 therapy, adjuvant radiotherapy and endocrine therapy, thus precluding the generalization of any conclusions. In particular, previous multimodality treatment may have influenced tumor biology and disease progression in this particular case. Finally, this case highlights possible caveats that may delay diagnosis of DCIS; in particular, the use of breast MRI and CNB instead of VAB in evaluating microcalcifications may lead to unacceptable delay of diagnosis with potentially serious medicolegal implications.

## Data Availability

The original contributions presented in this study are included in the article. Further inquiries can be directed to the corresponding author.
